# The genome sequence of the Buff Ermine, *Spilarctia lutea* (Hufnagel, 1766)

**DOI:** 10.12688/wellcomeopenres.19065.1

**Published:** 2023-02-21

**Authors:** Douglas Boyes, University of Oxford and Wytham Woods Genome Acquisition Lab, Darwin Tree of Life Barcoding collective, Wellcome Sanger Institute Tree of Life programme, Wellcome Sanger Institute Scientific Operations: DNA Pipelines collective, Tree of Life Core Informatics collective, Peter W. H. Holland, Darwin Tree of Life Consortium

**Affiliations:** 1 UK Centre for Ecology and Hydrology, Wallingford, Oxfordshire, UK; 2 University of Oxford, Oxford, Oxfordshire, UK

**Keywords:** Spilarctia lutea, the Buff Ermine, genome sequence, chromosomal, Lepidoptera

## Abstract

We present a genome assembly from an individual female *Spilarctia lutea* (the Buff Ermine; Arthropoda; Insecta; Lepidoptera; Erebidae). The genome sequence is 584.8 megabases in span. Most of the assembly is scaffolded into 32 chromosomal pseudomolecules, including the assembled Z and W sex chromosomes. The mitochondrial genome has also been assembled and is 15.4 kilobases in length. Gene annotation of this assembly on Ensembl identified 18,304 protein coding genes.

## Species taxonomy

Eukaryota; Metazoa; Ecdysozoa; Arthropoda; Hexapoda; Insecta; Pterygota; Neoptera; Endopterygota; Lepidoptera; Glossata; Ditrysia; Noctuoidea; Erebidae; Arctiinae; *Spilarctia*; *Spilarctia lutea* (Hufnagel, 1766) (NCBI:txid875881).

## Background

The Tiger moths, Footmen, Cinnabar moths and several Ermine moths comprise a taxonomic group of Lepidoptera characterised by distinctly ‘hairy’ larvae with long setae, an ultrasonic sound-producing organ on the thorax of the adult and, in many cases, bright colours and production of toxic chemicals. For many years these moths were placed in their own family, Arctiidae, but this is now considered a subfamily Arctiinae of the family Erebidae. The Buff Ermine *Spilarctia lutea* (sometimes placed in the genus Spilosoma) is a widely distributed example. Found across Europe and east into Russia and Mongolia, the species is common across England, Wales, Northern Ireland and western counties of Scotland, with scattered records from Ireland and central Scotland ( GBIF Secretariat, 2022; NBN Atlas, 2022).

The forewings of the adult are a sandy buff colour, often paler in females, with a broken diagonal lines of black spots running from the apex to the middle of trailing wing edge. The extent of the black markings can vary greatly, from being almost absent to being enlarged into pronounced streaks. The darker variants of *S. lutea* are uncommon in the wild, but have been studied in laboratory crosses that suggest several genetic loci contribute to wing patterning ( South, 1961). In all cases, the abdomen is bright yellow dorsally with black patches. The adult moths are conspicuous visually suggesting they may be aposematic. It has been noted they are unpalatable to some birds and their bodies have (relatively low) concentrations of pharmacologically-active substances ( Rothschild, 1963). An intriguing hypothesis, proposed by Miriam Rothschild, is that the Buff Ermine *S. lutea* is a mimic of the more poisonous White Ermine moth *S. lubricipeda*, sitting somewhere on a spectrum between Batesian and Müllerian mimicry ( Rothschild, 1963).

In the UK, adults of *S. lutea* are on the wing in June and July, laying eggs on the leaves of larval food plants including dandelion, dock, plantain and birch. If disturbed, the larvae drop to the ground and curl into a ring as a defence reaction, exposing their dense covering of hairs ( Brooks, 1991). There is one generation per year with the pupal stage overwintering.

A genome sequence of *S. lutea* will be useful for comparing the molecular basis of toxin production between species and for understanding the genetic basis of wing patterning.

## Genome sequence report

**Figure 1.**
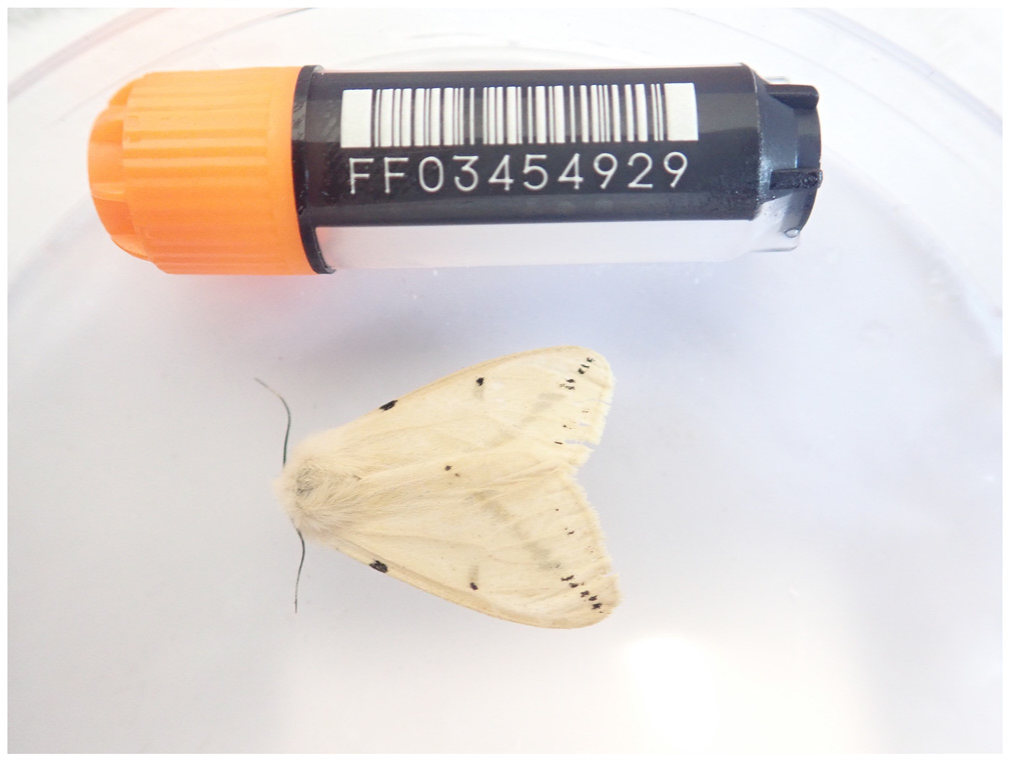
Image of the *Spilarctia lutea* (ilSpiLutu1) specimen used for genome sequencing.

The genome was sequenced from a female *Spilarctia lutea* specimen ( Figure 1) collected from Wytham Woods, UK (latitude 51.77, longitude –1.34) A total of 28-fold coverage in Pacific Biosciences single-molecule HiFi long reads and 116-fold coverage in 10X Genomics read clouds were generated. Primary assembly contigs were scaffolded with chromosome conformation Hi-C data. Manual assembly curation corrected 12 missing joins or mis-joins and removed one haplotypic duplication, reducing the scaffold number by 12.77%.

**Figure 2.**
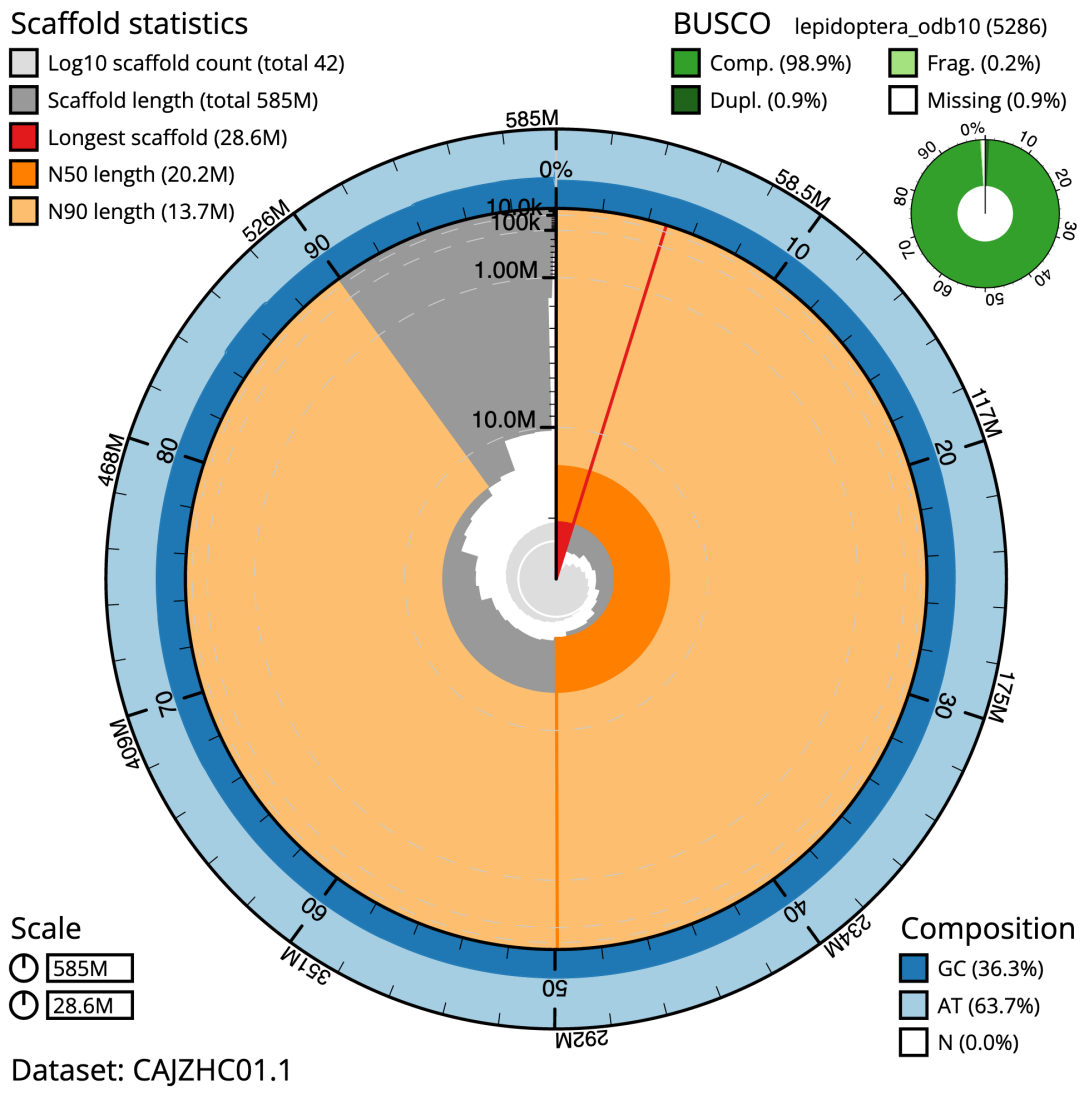
Genome assembly of *Spilarctia lutea*, ilSpiLutu1.1: metrics. The BlobToolKit Snailplot shows N50 metrics and BUSCO gene completeness. The main plot is divided into 1,000 size-ordered bins around the circumference with each bin representing 0.1% of the 584,787,352 bp assembly. The distribution of scaffold lengths is shown in dark grey with the plot radius scaled to the longest scaffold present in the assembly (28,626,442 bp, shown in red). Orange and pale-orange arcs show the N50 and N90 scaffold lengths (20,170,318 and 13,734,192 bp), respectively. The pale grey spiral shows the cumulative scaffold count on a log scale with white scale lines showing successive orders of magnitude. The blue and pale-blue area around the outside of the plot shows the distribution of GC, AT and N percentages in the same bins as the inner plot. A summary of complete, fragmented, duplicated and missing BUSCO genes in the lepidoptera_odb10 set is shown in the top right. An interactive version of this figure is available at https://blobtoolkit.genomehubs.org/view/ilSpiLutu1.1/dataset/CAJZHC01.1/snail.

The final assembly has a total length of 584.8 Mb in 41 sequence scaffolds with a scaffold N50 of 20.2 Mb ( Table 1). Most (99.98%) of the assembly sequence was assigned to 32 chromosomal-level scaffolds, representing 30 autosomes, and the Z and W sex chromosomes. Chromosome-scale scaffolds confirmed by the Hi-C data are named in order of size ( Figure 2– Figure 5; Table 2). The assembly has a BUSCO v5.3.2 ( Manni *et al.*, 2021) completeness of 98.9% (single 98.1%, duplicated 0.9%) using the lepidoptera_odb10 reference set ( *n* = 5286). While not fully phased, the assembly deposited is of one haplotype. Contigs corresponding to the second haplotype have also been deposited.

**Table 1.** Genome data for *Spilarctia lutea*, ilSpiLutu1.1.

Project accession data		
Assembly identifier	ilSpiLutu1.1	
Species	*Spilarctia lutea*	
Specimen	ilSpiLutu1	
NCBI taxonomy ID	875881	
BioProject	PRJEB46310	
BioSample ID	SAMEA7631557	
Isolate information	female ilSpiLutu1; abdomen (PacBio, Chromium), thorax (Hi-C, RNA-Seq)	
Assembly metrics *		*Benchmark*
Consensus quality (QV)	61.4	*≥ 50*
*k*-mer completeness	100%	*≥ 95%*
BUSCO **	C:98.9%[S:98.1%,D:0.9%], F:0.2%,M:0.9%,n:5,286	*C ≥ 95%*
Percentage of assembly mapped to chromosomes	99.98%	*≥ 95%*
Sex chromosomes	Z and W	*localised homologous pairs*
Organelles	Mitochondrial genome assembled	*complete single alleles*
Raw data accessions		
PacificBiosciences SEQUEL II	ERR6807997, ERR6939234	
10X Genomics Illumina	ERR6688470–ERR6688473	
Hi-C Illumina	ERR6688474	
PolyA RNA-Seq Illumina	ERR9434998	
Genome assembly		
Assembly accession	GCA_916048165.1	
*Accession of alternate haplotype*	GCA_916047975.1	
Span (Mb)	584.8	
Number of contigs	61	
Contig N50 length (Mb)	19.1	
Number of scaffolds	41	
Scaffold N50 length (Mb)	20.2	
Longest scaffold (Mb)	28.6	
Genome annotation		
Number of protein-coding genes	18,304	
Number of gene transcripts	18,498	

* Assembly metric benchmarks are adapted from column VGP-2020 of “Table 1: Proposed standards and metrics for defining genome assembly quality” from ( Rhie *et al.*, 2021).

** BUSCO scores based on the lepidoptera_odb10 BUSCO set using v5.3.2. C = complete [S = single copy, D = duplicated], F = fragmented, M = missing, n = number of orthologues in comparison. A full set of BUSCO scores is available at https://blobtoolkit.genomehubs.org/view/ilSpiLutu1.1/dataset/CAJZHC01.1/busco.

**Figure 3.**
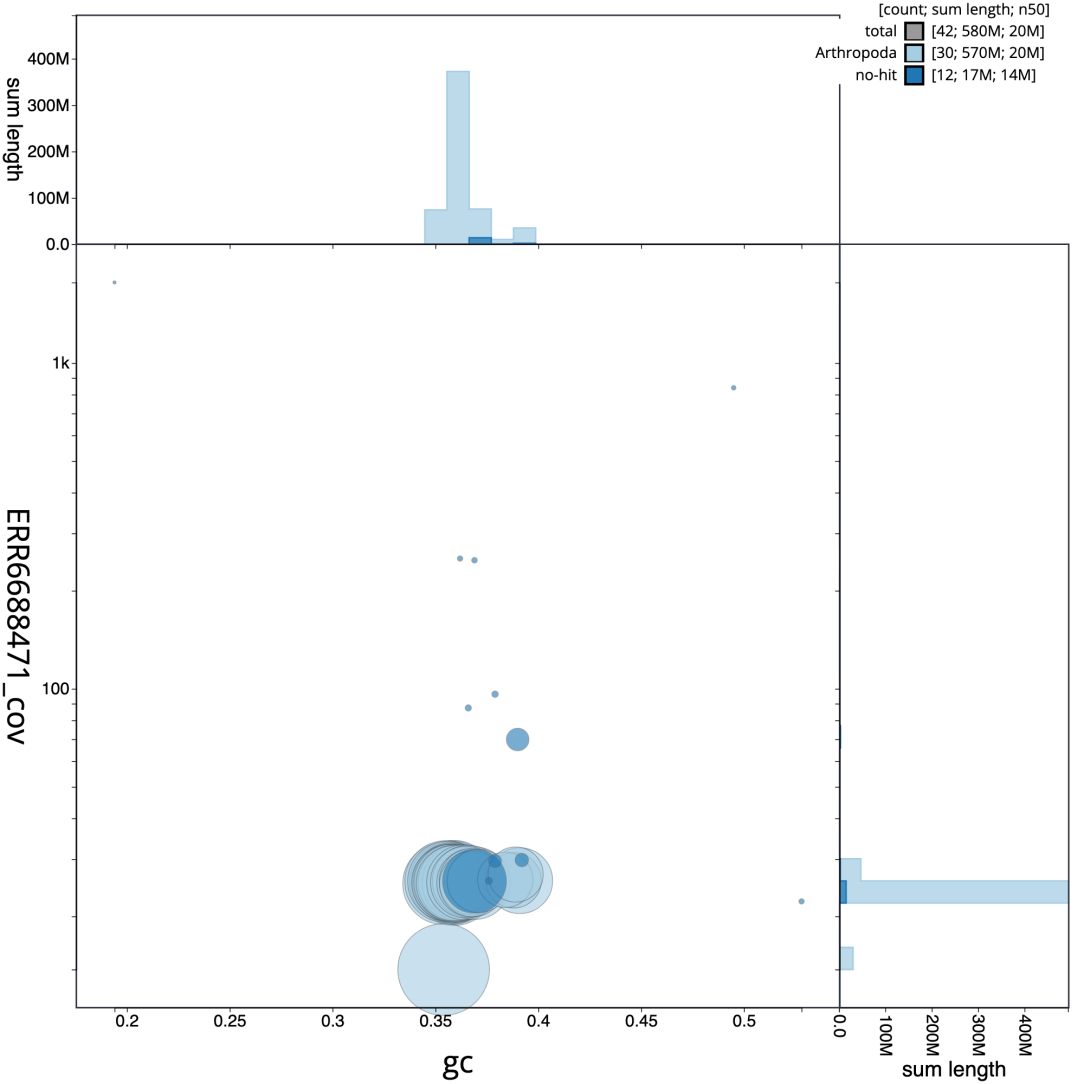
Genome assembly of *Spilarctia lutea*, ilSpiLutu1.1: GC coverage. BlobToolKit GC-coverage plot. Scaffolds are coloured by phylum. Circles are sized in proportion to scaffold length. Histograms show the distribution of scaffold length sum along each axis. An interactive version of this figure is available at https://blobtoolkit.genomehubs.org/view/ilSpiLutu1.1/dataset/CAJZHC01.1/blob.

**Figure 4.**
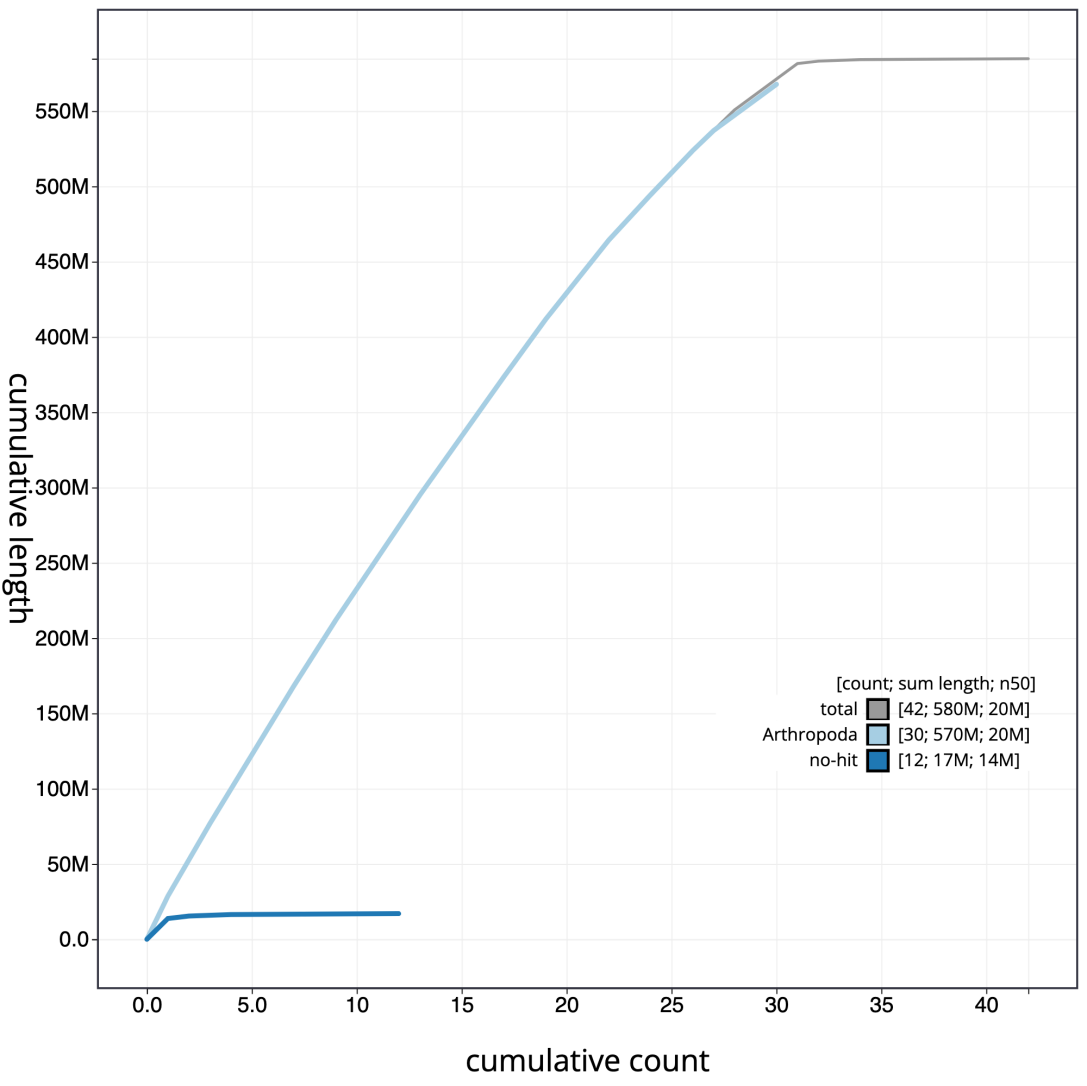
Genome assembly of *Spilarctia lutea*, ilSpiLutu1.1: cumulative sequence. BlobToolKit cumulative sequence plot. The grey line shows cumulative length for all scaffolds. Coloured lines show cumulative lengths of scaffolds assigned to each phylum using the buscogenes taxrule. An interactive version of this figure is available at https://blobtoolkit.genomehubs.org/view/ilSpiLutu1.1/dataset/CAJZHC01.1/cumulative.

**Figure 5.**
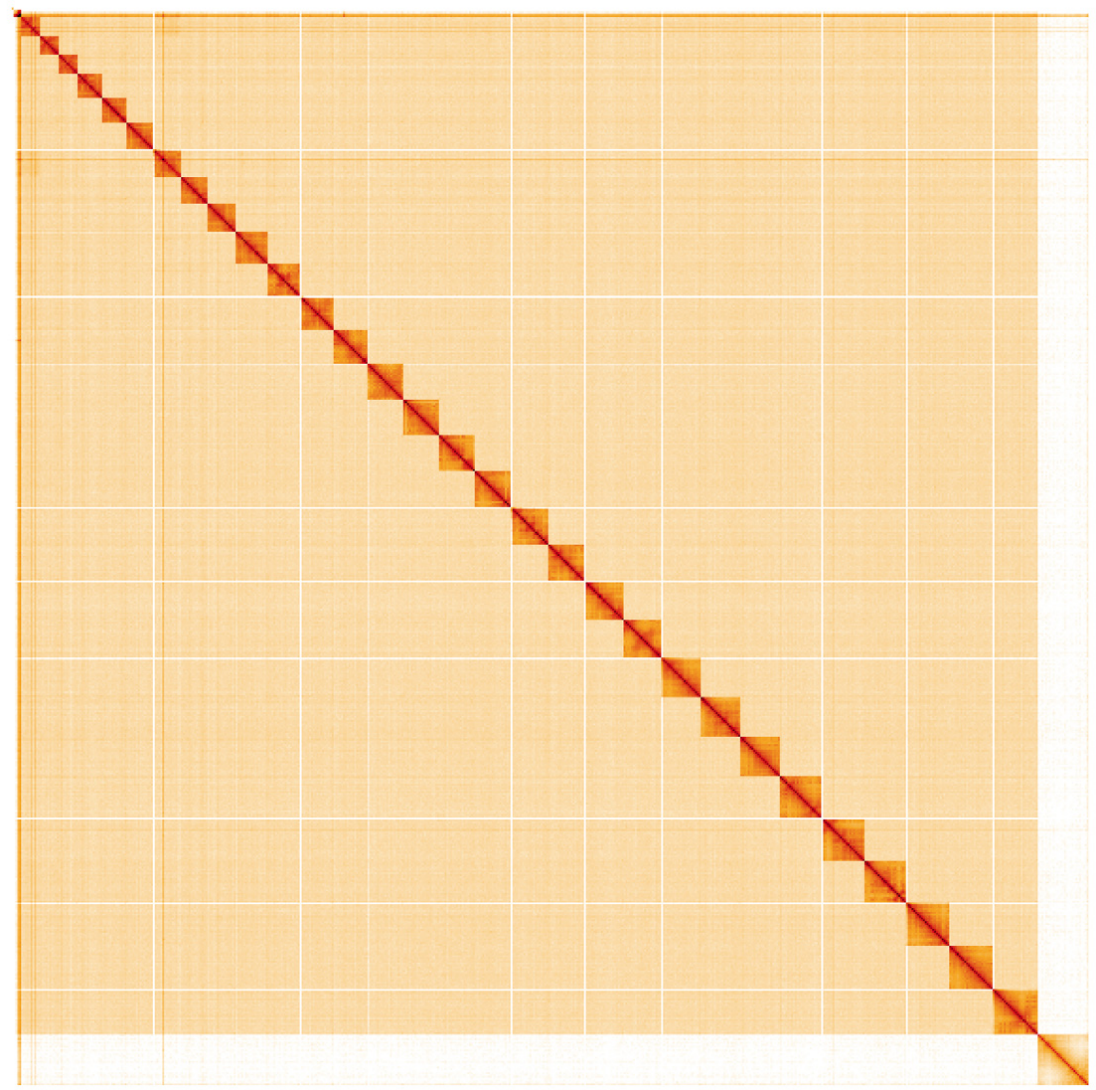
Genome assembly of *Spilarctia lutea*, ilSpiLutu1.1: Hi-C contact map. Hi-C contact map of the ilSpiLutu1.1 assembly, visualised using HiGlass. Chromosomes are shown in order of size from left to right and top to bottom. An interactive version of this figure may be viewed at https://genome-note-higlass.tol.sanger.ac.uk/l/?d=GwgyGmFqQMGAPGyNYPeTXw.

**Table 2.** Chromosomal pseudomolecules in the genome assembly of *Spilarctia lutea*, ilSpiLutu1.

INSDC accession	Chromosome	Size (Mb)	GC%
OU696471.1	1	24.14	35.9
OU696472.1	2	23.93	35.8
OU696473.1	3	23.13	35.8
OU696474.1	4	23.04	35.9
OU696475.1	5	22.64	35.4
OU696476.1	6	22.66	35.5
OU696477.1	7	21.99	35.7
OU696478.1	8	21.76	35.6
OU696479.1	9	21.08	35.8
OU696480.1	10	20.82	35.8
OU696481.1	11	20.81	35.9
OU696482.1	12	20.17	35.9
OU696483.1	13	19.78	35.7
OU696484.1	14	19.62	35.8
OU696485.1	15	19.62	36.2
OU696486.1	16	19.5	36
OU696487.1	17	19.26	35.9
OU696488.1	18	19.05	36.4
OU696489.1	19	17.78	36.9
OU696490.1	20	17.44	36.6
OU696491.1	21	17.32	36.5
OU696492.1	22	15.28	36.7
OU696493.1	23	14.91	36.8
OU696494.1	24	14.63	39.1
OU696495.1	25	14.6	36.8
OU696496.1	26	13.73	36.9
OU696497.1	27	13.4	37.1
OU696498.1	28	10.32	38.8
OU696499.1	29	10.29	38.4
OU696500.1	30	10.21	38.9
OU696501.1	W	1.63	39
OU696470.1	Z	28.63	35.4
OU696502.1	MT	0.02	19.5
-	unplaced	1.62	39.1

## Genome annotation report

The *S. lutea* genome assembly (GCA_916048165.1) was annotated using the Ensembl rapid annotation pipeline ( Table 1; https://rapid.ensembl.org/Spilarctia_lutea_GCA_916048165.1/). The resulting annotation includes 18,498 transcribed mRNAs from 18,304 protein-coding genes.

## Methods

### Sample acquisition and nucleic acid extraction

One female *S. lutea* specimen (ilSpiLutu1) was collected in Wytham Woods, Oxfordshire (biological vice-county: Berkshire), UK (latitude 51.77, longitude –1.34) on 22 May 2020. The specimen was caught in woodland habitat using a light trap. The specimen was collected and identified by Douglas Boyes (University of Oxford) and snap-frozen on dry ice.

DNA was extracted at the Tree of Life laboratory, Wellcome Sanger Institute (WSI). The ilSpiLutu1 sample was weighed and dissected on dry ice with thorax tissue set aside for RNA and Hi-C sequencing. Abdomen tissue was disrupted using a Nippi Powermasher fitted with a BioMasher pestle. High molecular weight (HMW) DNA was extracted using the Qiagen MagAttract HMW DNA extraction kit. Low molecular weight DNA was removed from a 20 ng aliquot of extracted DNA using 0.8X AMpure XP purification kit prior to 10X Chromium sequencing; a minimum of 50 ng DNA was submitted for 10X sequencing. HMW DNA was sheared into an average fragment size of 12–20 kb in a Megaruptor 3 system with speed setting 30. Sheared DNA was purified by solid-phase reversible immobilisation using AMPure PB beads with a 1.8X ratio of beads to sample to remove the shorter fragments and concentrate the DNA sample. The concentration of the sheared and purified DNA was assessed using a Nanodrop spectrophotometer and Qubit Fluorometer and Qubit dsDNA High Sensitivity Assay kit. Fragment size distribution was evaluated by running the sample on the FemtoPulse system.

RNA was extracted from thorax tissue of ilSpiLutu1 in the Tree of Life Laboratory at the WSI using TRIzol, according to the manufacturer’s instructions. RNA was then eluted in 50 μl RNAse-free water and its concentration assessed using a Nanodrop spectrophotometer and Qubit Fluorometer using the Qubit RNA Broad-Range (BR) Assay kit. Analysis of the integrity of the RNA was done using Agilent RNA 6000 Pico Kit and Eukaryotic Total RNA assay.

### Sequencing

Pacific Biosciences HiFi circular consensus and 10X Genomics read cloud DNA sequencing libraries were constructed according to the manufacturers’ instructions. Poly(A) RNA-Seq libraries were constructed using the NEB Ultra II RNA Library Prep kit. DNA and RNA sequencing was performed by the Scientific Operations core at the WSI on Pacific Biosciences SEQUEL II (HiFi), Illumina HiSeq 4000 (RNA-Seq) and NovaSeq 6000 (10X) instruments. Hi-C data were also generated from thorax tissue of ilSpiLutu1 using the Arima v2 kit and sequenced on the Illumina NovaSeq 6000 instrument.

### Genome assembly

Assembly was carried out with Hifiasm ( Cheng *et al.*, 2021) and haplotypic duplication was identified and removed with purge_dups ( Guan *et al.*, 2020). One round of polishing was performed by aligning 10X Genomics read data to the assembly with Long Ranger ALIGN, calling variants with freebayes ( Garrison & Marth, 2012). The assembly was then scaffolded with Hi-C data ( Rao *et al.*, 2014) using SALSA2 ( Ghurye *et al.*, 2019). The assembly was checked for contamination as described previously ( Howe *et al.*, 2021). Manual curation was performed using HiGlass ( Kerpedjiev *et al.*, 2018) and Pretext ( Harry, 2022). The mitochondrial genome was assembled using MitoHiFi ( Uliano-Silva *et al.*, 2022), which performed annotation using MitoFinder ( Allio *et al.*, 2020). The genome was analysed and BUSCO scores were generated within the BlobToolKit environment ( Challis *et al.*, 2020). Table 3 contains a list of all software tool versions used, where appropriate.

**Table 3.** Software tools and versions used.

Software tool	Version	Source
BlobToolKit	3.5.2	Challis *et al.*, 2020
freebayes	1.3.1-17- gaa2ace8	Garrison & Marth, 2012
Hifiasm	0.15.1	Cheng *et al.*, 2021
HiGlass	1.11.6	Kerpedjiev *et al.*, 2018
Long Ranger ALIGN	2.2.2	https://support.10xgenomics. com/genome-exome/software/ pipelines/latest/advanced/ other-pipelines
MitoHiFi	2	Uliano-Silva *et al.*, 2022
PretextView	0.2	Harry, 2022
purge_dups	1.2.3	Guan *et al.*, 2020
SALSA	2.2	Ghurye *et al.*, 2019

### Genome annotation

The BRAKER2 pipeline ( Brůna *et al.*, 2021) was used in the default protein mode to generate annotation for the *Spilarctia lutea* assembly (GCA_916048165.1) in Ensembl Rapid Release.

### Ethics and compliance issues

The materials that have contributed to this genome note have been supplied by a Darwin Tree of Life Partner. The submission of materials by a Darwin Tree of Life Partner is subject to the Darwin Tree of Life Project Sampling Code of Practice. By agreeing with and signing up to the Sampling Code of Practice, the Darwin Tree of Life Partner agrees they will meet the legal and ethical requirements and standards set out within this document in respect of all samples acquired for, and supplied to, the Darwin Tree of Life Project. All efforts are undertaken to minimise the suffering of animals used for sequencing. Each transfer of samples is further undertaken according to a Research Collaboration Agreement or Material Transfer Agreement entered into by the Darwin Tree of Life Partner, Genome Research Limited (operating as the Wellcome Sanger Institute), and in some circumstances other Darwin Tree of Life collaborators.

## Data Availability

European Nucleotide Archive: *Spilarctia lutea* (Buff Ermine). Accession number PRJEB46310; https://identifiers.org/ena.embl/PRJEB46310. ( Wellcome Sanger Institute, 2021) The genome sequence is released openly for reuse. The *Spilarctia lutea* genome sequencing initiative is part of the Darwin Tree of Life (DToL) project. All raw sequence data and the assembly have been deposited in INSDC databases. Raw data and assembly accession identifiers are reported in Table 1.

## Author information

Members of the University of Oxford and Wytham Woods Genome Acquisition Lab are listed here: https://doi.org/10.5281/zenodo.4789928.

Members of the Darwin Tree of Life Barcoding collective are listed here: https://doi.org/10.5281/zenodo.4893703.

Members of the Wellcome Sanger Institute Tree of Life programme are listed here: https://doi.org/10.5281/zenodo.4783585.

Members of Wellcome Sanger Institute Scientific Operations: DNA Pipelines collective are listed here: https://doi.org/10.5281/zenodo.4790455.

Members of the Tree of Life Core Informatics collective are listed here: https://doi.org/10.5281/zenodo.5013541.

Members of the Darwin Tree of Life Consortium are listed here: https://doi.org/10.5281/zenodo.4783558.
