# Supplementary material for: The genome sequence of the Buff Ermine, Spilarctia lutea (Hufnagel, 1766)
Source: Wellcome Open Res. 2023 Feb 21;8:92. [Version 1] doi: 10.12688/wellcomeopenres.19065.1 (PMC10444995; doi:10.12688/wellcomeopenres.19065.1)
Supplement: Peer review history

# Reviewer report: Reviewer response for version 1

**DOI:** 10.21956/wellcomeopenres.21138.r64611

**Published:** 2023-09-11

**Authors:** Renata Da Rosa

**Affiliations:** 1 Universidade Estadual de Londrina, Londrina, State of Paraná, Brazil

The paper presents a genome assembly from a individual female of *Spilarctia lutea.* The study was well done, and the results were clearly explained. The figures and tables are very clear, the images provided are resolutive and the captions are extremely informative. Downloadable files are available in the repository. The techniques used were sufficient for that proposed by the authors. I recommend the indexing of the data paper.

Are sufficient details of methods and materials provided to allow replication by others?

Yes

Is the rationale for creating the dataset(s) clearly described?

Yes

Are the datasets clearly presented in a useable and accessible format?

Yes

Are the protocols appropriate and is the work technically sound?

Yes

Reviewer Expertise:

Animal genetics

I confirm that I have read this submission and believe that I have an appropriate level of expertise to confirm that it is of an acceptable scientific standard.

# Reviewer report: Reviewer response for version 1

**DOI:** 10.21956/wellcomeopenres.21138.r64914

**Published:** 2023-08-22

**Authors:** Paula Escuer

**Affiliations:** 1 University of Neuchâtel, Neuchâtel, Canton of Neuchâtel, Switzerland

The authors presented the chromosome level genome sequence of a female individual of the Buff Ermine ( *Spilarctia lutea*). The final assembly shows a total length of 584.8 Mb in 41 scaffolds with a scaffold N50 of 20.2 Mb. It is assembled with 28-fold coverage of HiFi long reads and 116-fold coverage of 10X Genomics read clouds and chromosome conformation Hi-C data. Mitochondrial assembly is also sequenced and available. The 99.98% of the assembly sequence is distributed in 32 chromosomal-level scaffolds, representing 30 autosomes plus the Z and W sex chromosomes. The BUSCO pipeline indicated a high completeness of the genome with 99.98% of complete genes found, additional descriptive analyses are indicated in Table 1. Genome annotation predicted 18,304 protein-coding genes. Standard bioinformatic pipelines were performed and software versions are indicated in Table 3. This high quality assembly is a great contribution that will allow uncovering a wide range of questions related to Lepidoptera genomics and evolution.

 My only concern to mention would be that they sequenced RNA-Seq data but didn’t include it when using the BRAKER pipeline to improve the certainty of the prediction, or at least it is not mentioned in the methods.

Are sufficient details of methods and materials provided to allow replication by others?

Yes

Is the rationale for creating the dataset(s) clearly described?

Yes

Are the datasets clearly presented in a useable and accessible format?

Yes

Are the protocols appropriate and is the work technically sound?

Yes

Reviewer Expertise:

Arthropoda genomics and transcriptomics, genome sequencing and annotation

I confirm that I have read this submission and believe that I have an appropriate level of expertise to confirm that it is of an acceptable scientific standard.

# Reviewer report: Reviewer response for version 1

**DOI:** 10.21956/wellcomeopenres.21138.r64612

**Published:** 2023-08-08

**Authors:** Zhaofu Yang

**Affiliations:** 1 Northwest A&F University, Yangling, Shaanxi, China

This paper describes the genome assembly of *Spilarctia lutea* and provides important information about its size (~584 Mb) and scaffolding (32 chromosomes). The mitochondrial genome has also been assembled. The methods for the sequencing and assembly used are appropriate and technically sound. This assembly provides valuable genomic resource for further investigations in the evolutionary pattern of Lepidoptera.

 The distributional range of *Spilarctia lutea* should be rectified as it has been reported in Northeast China. The generic placement of this species is likely confusing because it subordinate to the genus *Spilosoma Butler* 1875 in LepIndex ( https://www.nhm.ac.uk/our-science/data/lepindex/search/). I suggest authors consult some folks working on this group to confirm its status.

Are sufficient details of methods and materials provided to allow replication by others?

Yes

Is the rationale for creating the dataset(s) clearly described?

Yes

Are the datasets clearly presented in a useable and accessible format?

Yes

Are the protocols appropriate and is the work technically sound?

Yes

Reviewer Expertise:

Systematics, DNA barcoding, Lepidoptera, Taxonomy

I confirm that I have read this submission and believe that I have an appropriate level of expertise to confirm that it is of an acceptable scientific standard.
